# A Case of Diffuse Leptomeningeal Glioneuronal Tumor Misdiagnosed as Chronic Tuberculous Meningitis without Brain Biopsy

**DOI:** 10.1155/2018/1391943

**Published:** 2018-07-02

**Authors:** Jung koo Lee, Hak-cheol Ko, Jin-gyu Choi, Youn Soo Lee, Byung-chul Son

**Affiliations:** ^1^Department of Neurosurgery, Seoul St. Mary's Hospital, College of Medicine, The Catholic University of Korea, Seoul, Republic of Korea; ^2^Department of Hospital Pathology, Seoul St. Mary's Hospital, College of Medicine, The Catholic University of Korea, Seoul, Republic of Korea; ^3^Catholic Neuroscience Institute, College of Medicine, The Catholic University of Korea, Seoul, Republic of Korea

## Abstract

Here we report a rare case of diffuse leptomeningeal glioneuronal tumor (DLGNT) in a 62-year-old male patient misdiagnosed as having tuberculous meningitis. Due to its rarity and radiologic findings of leptomeningeal enhancement in the basal cisterns on magnetic resonance imaging (MRI) similar to tuberculous meningitis, DLGNT in this patient was initially diagnosed as communicating hydrocephalus from tuberculous meningitis despite absence of laboratory findings of tuberculosis. The patient's symptoms and signs promptly improved after a ventriculoperitoneal shunting surgery followed by empirical treatment against tuberculosis. Five years later, mental confusion and ataxic gait developed in this patient again despite well-functioning ventriculoperitoneal shunt. Aggravation of leptomeningeal enhancement in the basal cisterns was noted in MRI. An additional course of antituberculosis medication with steroid was started without biopsy of the brain. Laboratory examinations for tuberculosis were negative again. After four months of improvement, his mental confusion, memory impairment, dysphasia, and ataxia gradually worsened. A repeated MRI of the brain showed further aggravation of leptomeningeal enhancement in the basal cisterns. Biopsy of the brain surface and leptomeninges revealed a very rare occurrence of DLGNT. His delayed diagnosis of DLGNT might be due to prevalence of tuberculosis in our country, similarity in MRI finding of prominent leptomeningeal enhancement in the basal cisterns, and extreme rarity of DLGNT in the elderly. DLGLT should be considered in differential diagnosis of medical conditions presenting as communicating hydrocephalus with prominent leptomeningeal enhancement. A timely histologic diagnosis through a leptomeningeal biopsy of the brain and spinal cord in case of unusual leptomeningeal enhancement with uncertain laboratory findings is essential because cytologic examination of the cerebrospinal fluid in DLGNT is known to be negative.

## 1. Introduction

Glioneuronal tumors are a group of primary brain neoplasms of relatively recent acquisition in the World Health Organization (WHO) classification of central nervous system (CNS) tumors [1]. In the literature, they have been described in a variety of similar terms, e.g., DLGNT or disseminated oligodendroglial-like leptomeningeal tumor of childhood [2, 3]. They mostly present as diffuse leptomeningeal diseases in children and adolescents. Their histologic characteristics include monomorphic clear cell glial morphology reminiscent of oligodendroglioma, although they often express synaptophysin in addition to OLIG2 and S-100 [2, 3]. The hallmark of neuroradiological appearance of diffuse leptomeningeal glioneuronal tumor (DLGNT) is prominent leptomeningeal enhancement with or without communicating hydrocephalus [1]. On T1 gadolinium-enhanced images, a thick and diffuse leptomeningeal enhancement on the surface of brain and basal cisterns similar to that described in tuberculous meningitis has been documented in all DLGNT patients [1].

We present an extremely rare occurrence of DLGNT in a 62-year-old male patient. Despite no evidence of tuberculous meningitis, consideration of typical MRI findings of leptomeningeal enhancement in basal cisterns associated with hydrocephalus and prevalence of tuberculosis led to a tentative diagnosis of tuberculous meningitis. A ventriculoperitoneal shunt and medical treatment for tuberculosis were performed without invasive brain biopsy. Indeed, the diagnosis of tuberculous meningitis is often difficult because its clinical features are not very specific. Detection of* Mycobacterium tuberculosis* in cerebrospinal fluid (CSF) by acid-fast staining, culture, or DNA analysis with polymerase chain reaction (PCR) has low sensitivity. The current case highlights the importance of histologic confirmation through brain biopsy for cases presenting leptomeningeal enhancement in the basal cistern in MRI with equivocal laboratory examinations to explain the etiology.

## 2. Case Presentation

**Figure 1:**
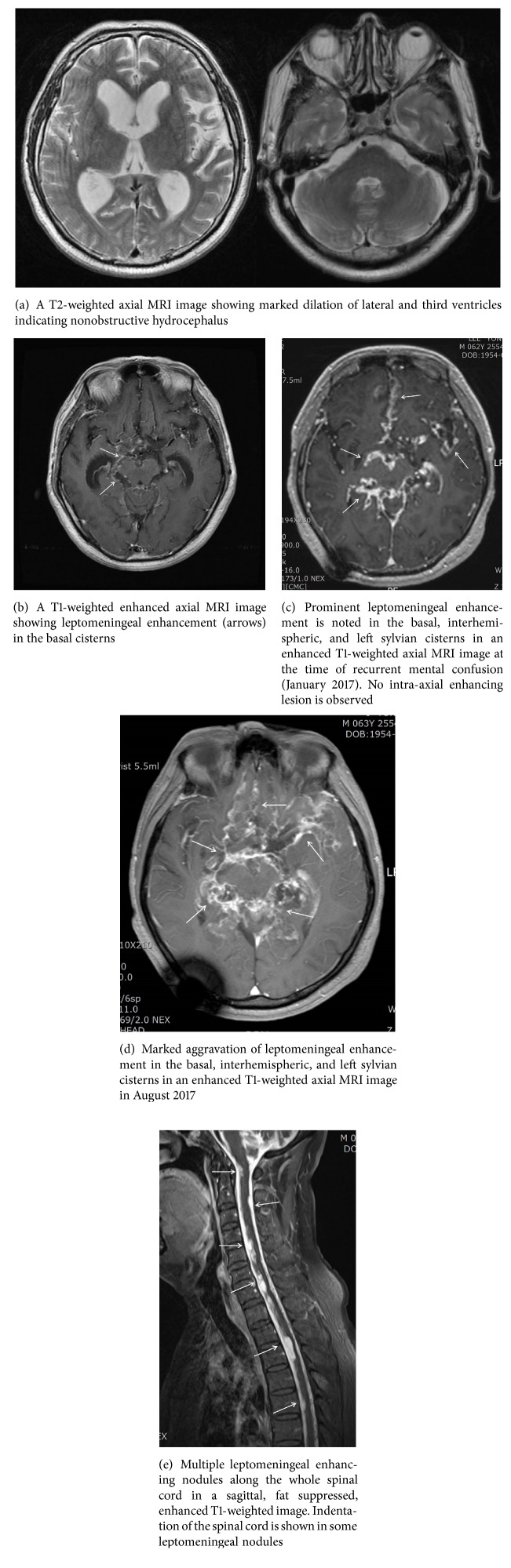
Magnetic resonance imaging (MRI) findings of communicating hydrocephalus and leptomeningeal enhancement in the basal cistern at the time of initial manifestation of mental confusion (2012).

A 62-year-old male patient presented with progressive worsening of mental function, dysphasia, and ataxic gait in the last six months. Five years prior to presentation (in August 2012), he was diagnosed with communicating hydrocephalus possibly caused by tuberculous meningoencephalitis because of mental confusion and gait disturbance. He underwent a ventriculoperitoneal shunt surgery in one hospital. His mental confusion and gait disturbance immediately improved following the ventriculoperitoneal shunt. Results of CSF study were negative for tuberculosis. However, a provisional diagnosis of communicating hydrocephalus caused by tuberculous meningitis was made based on MRI findings of leptomeningeal enhancement in the basal cisterns (Figures 1(a) and 1(b)). He had been treated with antituberculosis medication for the following six months after the shunting operation. After shunting and medical treatment, he returned to his work. He had been followed-up regularly every six months at that hospital. His physical and mental conditions were stable. He experienced no difficulty in work or daily activities.

Six months prior to the present presentation (December 2016), slurred speech and mental confusion with intermittent disorientation to time and place developed within several days. CSF analysis and MRI of the brain were performed. CSF analysis showed white blood cell (WBC) count of 9 cells/*μ*L, red blood cell count of 33,000 cell/*μ*L, protein level of 4228 mg/dL, lactic dehydrogenase (LDH) level of 224 mg/dL, and glucose level of 130 mg/dL. MRI of the brain showed multiple linear and nodular leptomeningeal enhancing lesions scattered in basal and left sylvian cisterns (Figure 1(c)). The extent of leptomeningeal enhancement in basal cisterns was markedly increased compared to that in MRI examination done in 2012. The size of the ventricle was small, indicating that shunt malfunction did not occur. There was no abnormal spike activity in his electroencephalography (EEG) except intermittent slow wave in his left frontocentral area. Under an impression of aggravation of tuberculosis meningitis, he was referred to our hospital (January 2017).

The patient's consultation in the Department of Infectious Medicine was carried out for aggravation of tuberculous meningitis/encephalitis. The doctor in neurology thought that tuberculous meningitis aggravated again. For possibility of drug-resistant tuberculosis, four-drug regimen (isoniazid 75 mg, rifampicin 150 mg, pyrazinamide 400 mg, and ethambutol 300 mg; tubes tab 4 times a day for 2 months followed by isoniazide and rifampicin for 7 months) against tuberculosis was used. Beside antituberculosis medications, steroid was prescribed. The patient's mental confusion, dysphasia, and irritability progressively improved over the course of one month at the outpatient clinic. He returned to his usual life again. He was able to work in his previous job without apparent complications.

His mental confusion and dysphasia accompanying gait disturbance gradually developed again within four months (June 2017), leading to reevaluation of the brain by MRI. There was no fever or signs of meningeal irritation in neurologic evaluation. MRI of the brain surface revealed extensive progression of diffuse leptomeningeal enhancement in the basal and left sylvian cisterns (Figure 1(d)). No intraparenchymal enhancing lesion was noted. Hydrocephalic change was not shown either. CSF examination showed WBC count of 110 cells/*μ*L (lymphocyte 70%, macrophage 7%, and neutrophils 3%), red blood cell count of 7200 cell/*μ*L, protein level of 4272 mg/dL, and glucose level of 102 mg/dL. Levels of erythrocyte sedimentation rate (ESR) and C-reactive protein (CRP) were 4 mm/hr and 0.05 mg/dl, respectively. Levels of adenosine deaminase (ADA) and immunoglobulin G were 8.0 IU/L and 901 mg/dl, respectively. Results of CSF culture for toxoplasmosis, fungus, cryptococcus, and herpes simplex virus were all negative. Gram-staining revealed many WBC without microorganism. Polymerase chain reactions (PCR) of the CSF against* Mycobacterium tuberculosis*, herpes simplex virus, varicella zoster, enterovirus, and Epstein-Barr virus were all negative. Culture for acid-fast bacilli (AFB) did not show any growth until eight weeks after incubation. For possibility of leptomeningeal metastasis, biopsy of the brain, and leptomeninges was requested.

**Figure 2:**
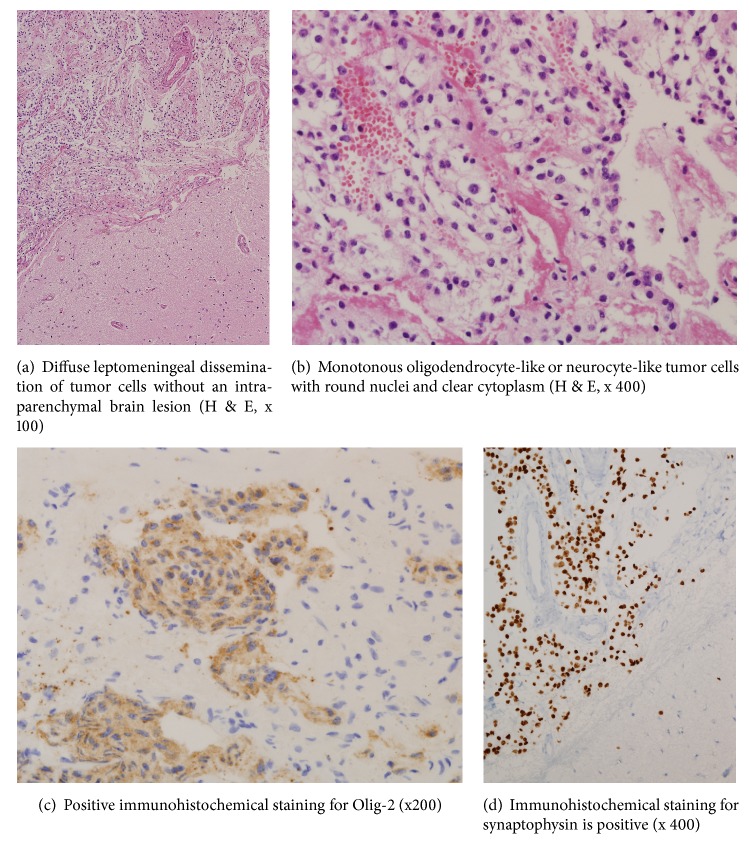
Histopathologic examination of disseminated leptomeningeal glioneuronal tumor.

Biopsy of the brain surface and leptomeninges was performed on the left frontal cortex and sylvian fissure proceeded by a small frontotemporal craniotomy. Postoperative course was uneventful. Histologic diagnosis revealed DLGNT without intraparenchymal brain lesion (Figure 2(a)). Monotonous oligodendrocyte-like or neurocyte-like tumor cells with round nuclei and clear cytoplasm were found (Figure 2(b)). Mitosis, microvascular proliferation, and necrosis were not evident. Immunohistochemical stainings for Olig-2 and synaptophysin were positive (Figures 2(c) and 2(d)). Those for CD68, isocitrate dehydrogenase- (IDH-) 1, glial fibrillary acidic protein (GFAP), and neurofilament were negative. Ki67 proliferative index was low (5%). PCR for O^6^-methylguanine-DNA-methyltransferase (MGMT) methylation was positive. However, 1p19q codeletion was not detected by interphase fluorescent in situ hybridization (FISH). Methenamine-silver and PAS staining for fungal organism, Ziehl-Neelsen staining, and PCR for* Mycobacterium tuberculosis* were all negative. After histologic diagnosis of DLGNT, MRI of the whole spine was subsequently performed in order to detect further leptomeningeal spread. MRI showed multiple leptomeningeal enhancing nodules displaying high signal intensity on T2-weighted images (Figure 1(e)), disseminating along the whole spinal cord without intramedullary lesion. With a final diagnosis of DLGNT by invasive brain biopsy, medical records and imaging results were thoroughly reviewed again. PCV (Procarbazine, CCNU, and Vincristine) chemotherapy and radiation therapy of the craniospinal axis were planned. The patient's condition gradually deteriorated with apparent worsening of severe memory impairment, disorientation, and gait ataxia.

## 3. Discussion

### 3.1. Leptomeningeal Enhancement and Tuberculous Meningitis

Contrast material enhancement for cross-sectional imaging has been used since the mid-1970s for computed tomography and the mid-1980s for MRI [4]. Knowledge of patterns of contrast enhancement has facilitated clinical and radiologic differential diagnosis. Extra-axial enhancement in the CNS may be classified as either pachymeningeal (dura mater, thick meninges) or leptomeningeal (pia and arachnoid, skinny meninges). Enhancement of the pia mater or enhancement extending into the subarachnoid spaces of the sulci and cisterns is leptomeningeal enhancement. It is also called “pial or pia-arachnoid enhancement”. Leptomeningeal enhancement is usually associated with meningitis and meningoencephalitis that might be bacterial, viral, or fungal. The primary mechanism of this enhancement is breakdown of the blood-brain barrier without angiogenesis. The subarachnoid space is infiltrated with inflammatory cells. The permeability in the meninges may increase due to bacterial glycoproteins released into the subarachnoid space [4]. Neoplasms may spread into the subarachnoid space and produce enhancement of the brain surface and subarachnoid space, a pathologic process often called “carcinomatous meningitis”. Both primary tumors (medulloblastoma, ependymoma, glioblastoma, and oligodendroglioma) and secondary tumors (e.g., lymphoma and breast cancer) may spread through the subarachnoid space. Neoplasmic leptomeningeal enhancement often produces thick, lumpy, or nodular enhancement, similar to fungal meningitis.

Tuberculosis has shown resurgence in nonendemic populations in recent years due to increased migration and endemic human immunodeficiency virus [5]. Although the thorax is most frequently involved, tuberculosis may involve any organ systems. Its involvement in the CNS is seen in approximately 5% of patients with tuberculosis [6]. CNS tuberculosis can manifest in a variety of forms, including tuberculous meningitis, tuberculomas, tuberculous abscesses, tuberculous cerebritis, and miliary tuberculosis. Among these, tuberculous meningitis is the most common manifestation of CNS involvement across all age groups [7]. It is usually due to hematogenous spread. However, it can be secondary to rupture of a Rich focus or direct extension from CSF infection [5–7]. Its typical radiographic finding is abnormal meningeal enhancement usually most pronounced in basal cisterns, although meningeal involvement at some degree within the sulci over the cerebral convexities and in sylvian fissures is also seen in many cases [5–8]. Early diagnosis is important to reduce morbidity and mortality because delayed treatment is associated with severe morbidity. Unfortunately, history of infection or exposure to tuberculosis may or may not present in tuberculosis patients. Evidence of active tuberculosis is present in less than 50% of cases. Furthermore, clinical and radiologic features of tuberculosis may mimic those of many other diseases.

### 3.2. Delayed Diagnosis of DLGNT

The diagnosis of DLGLT was delayed in the current case. The patient initially presented with altered mentality with MRI findings of leptomeningeal enhancement of basal cisterns and communicating hydrocephalus. Characteristic basal meningeal inflammation resulting in leptomeningeal enhancement in basal cisterns is the most typical feature of gadolinium-enhanced MR imaging of tuberculous meningitis. The most common complication of tuberculous meningitis is communicating hydrocephalus caused by blockage of basal cisterns due to inflammatory exudates [4, 8]. In addition, tuberculosis is still prevalent and multidrug-resistant tuberculosis is one of the major medical concerns in our country. Difficulty in establishing a diagnosis of tuberculous meningitis might have also contributed to the diagnostic error. A positive mycobacterial culture in the CSF remains the gold standard in the diagnosis of tuberculous meningitis. However, CSF acid-fast bacilli have been identified in less than 10% of cases. Mycobacteria culture positivity ranges from 50% to 75% after 8 weeks, an unacceptable length of time for the diagnosis of tuberculosis in making treatment decision [9, 10]. In the current case, real-time polymerase chain reaction (PCR) and mycobacterial cultures from sputum and CSF were negative. Despite absence of laboratory data supporting tuberculosis, the current case was treated with ventriculoperitoneal shunt and antituberculosis chemotherapy under clinical impression of tuberculous meningitis, complicated with hydrocephalus. In addition, radiological imaging study was not conducted to evaluate the efficacy of antituberculous treatment and resolution of leptomeningeal enhancement in the basal cisterns. According to medical standards, just clinical follow-up visits were scheduled and no control of clearance of the supposed tuberculous lesions was carried out.

Another reason for delayed diagnosis for this case might be an extreme rarity of DLGLT. Indeed, the patient did not show any past history or symptoms indicative of glial brain tumor. CSF cytology result was negative. Without an invasive brain biopsy including leptomeninges, it is hard to figure out such a rare DLGNT. The number of reported cases of DLGNT is less than 100 worldwide since the first report of the largest series of 36 patients by Rodrigues et al. in 2012 [2]. DLGNT has been mostly reported in children less than 10 years of age, although some of them have occurred in middle aged patients [1, 2, 11–17]. Prior to this report, there have been several case series and case reports published that might have the same entity. They were variably described as diffuse leptomeningeal glioneuronal tumor [1], superficially disseminated glioma in children [12], or diffuse leptomeningeal oligodendrogliomatosis [13–15]. These tumors including DLGNT are characterized radiologically by leptomeningeal enhancement on MRI usually involving basal cisterns and the spinal cord.

While various CNS tumors show diffuse leptomeningeal spread, Perilongo et al. [16] and Gardiman et al. [1] have reported a possibly novel entity of low-grade pediatric tumors with extensive leptomeningeal dissemination without a large solid component. It cannot be placed in the 2007 version of WHO classification of CNS tumors [18]. Histologically, these tumors are characterized as monomorphous oligoid tumor cells with round oval nuclei. Gardimann et al. [1] have suggested a “glioneuronal component” of these tumors and proposed a term of “diffuse leptomeningeal glioneuronal tumor”. Although clinical presentation and course in patients with DLGNT are still largely unknown [17], most patients present an acute onset of signs and symptoms of raised intracranial pressure caused by communicating hydrocephalus necessitating extraventricular drainage or ventriculoperitoneal shunt [2, 17]. Most patients initially received antibiotic treatment for suspected meningeal infection and MRI showed typical findings of leptomeningeal enhancement, similar to reactive postinfectious changes. Extensive CSF examinations including virology, inflammation, and tumor markers (beta-HCG, AFF, and PLAP) are required.

If the CSF specimen is insufficient to confirm diagnosis, a prompt and open arachnoid biopsy is necessary to confirm the diagnosis. An aggressive behavior has been reported in 38% of cases. However, most tumors seem to show periods of stability or slow progress [2]. In the current case, an open leptomeningeal biopsy was requested for recurrence of mental confusion with MRI findings of extensive aggravation of leptomeningeal enhancement of the brain. MRI of the spinal cord was requested according to extensive leptomeningeal enhancement of basal cisterns and posterior fossa. Treatment and clinical outcomes of DLGNT are not defined yet [11]. It is known that up to a third of patients may die of DLGNT, although other outcomes are not well reported yet [11]. Chemotherapy and radiotherapy have been tried. However, their effects on the outcome of patients with DLGNT have not been firmly validated yet.

## 4. Conclusion

We report an extremely rare occurrence of DLGNT in an elderly patient. His diagnosis was delayed and he was misdiagnosed as having a communicating hydrocephalus caused by tuberculous meningitis. Diagnostic error seems to be caused by difficulty in establishing a diagnosis of tuberculous meningitis, prevalence of tuberculosis in Asian country, similarity in MRI finding of leptomeningeal enhancement in basal cisterns, and an extreme rarity of DLGNT in the elderly. Although invasive, a prompt open biopsy of leptomeninges of the brain and spinal cord should be performed in case of diagnostic uncertainty in patients with typical findings of extensive leptomeningeal enhancement in basal cisterns. DLGLT should be listed in differential diagnosis of diseases causing leptomeningeal enhancement.
